# Cardiology–cardiothoracic subspeciality training in South Africa: a position paper of the South Africa Heart Association

**DOI:** 10.5830/CVJA-2016-063

**Published:** 2016

**Authors:** Karen Sliwa, Liesl Zühlke, Robert Kleinloog, Anton Doubell, Iftikhar Ebrahim, Mohammed Essop, David Kettles, David Jankelow, Sajidah Khan, Eric Klug, Andrew Thornton, Sandrine Lecour, David Marais, Martin Mpe, Mpiko Ntsekhe, Ashley Chin, Les Osrin, Francis Smit, Adriaan Snyders, Jean Paul Theron, Nico van der Merwe, Erika Dau, Andrew Sarkin

**Affiliations:** Hatter Institute for Cardiovascular Research in Africa, MRC Inter-University Cape Heart Group, Department of Medicine, Faculty of Health Sciences, University of Cape Town; Soweto Cardiovascular Research Unit, University of the Witwatersrand, Johannesburg, South Africa; Departments of Paediatric Cardiology and Medicine, Faculty of Health Sciences, University of Cape Town, South Africa; Cardiothoracic Surgery, Ethekwini Hospital and Heart Centre, Durban, South Africa; Division of Cardiology, Faculty of Medicine and Health Sciences, Stellenbosch University and Tygerberg Hospital, Tygerberg, South Africa; Netcare Unitas Hospital, Lyttleton Manor, Pretoria, South Africa; Division of Cardiology, Baragwanath Hospital and Faculty of Health Sciences, University of the Witwatersrand, Johannesburg, South Africa; St Dominic’s Hospital, and Frere Hospital, East London, South Africa; Netcare Linksfield Hospital, Linksfield West, Johannesburg, South Africa; Department of Cardiology, Faculty of Health Sciences, University of KwaZulu Natal, Durban, South Africa; Netcare Sunninghill Hospital, Sunninghill, Johannesburg, South Africa; Netcare Sunninghill Hospital, Sunninghill, Johannesburg, South Africa; Hatter Institute for Cardiovascular Research in Africa, Department of Medicine, Faculty of Health Sciences, University of Cape Town, South Africa; Division of Chemical Pathology, Faculty of Health Sciences, University of Cape Town, South Africa; Mediclinic Heart Hospital, Arcadia, Pretoria, South Africa; Department of Cardiology, Faculty of Health Sciences, University of Cape Town, South Africa; Department of Cardiology, Faculty of Health Sciences, University of Cape Town, South Africa; Zuid-Afrikaans Hospital, Muckleneuk and Department of Cardiology, Steve Biko Academic Hospital, University of Pretoria, Pretoria, South Africa; Department of Cardiothoracic Surgery, Faculty of Health Sciences, University of the Free State, Bloemfontein, South Africa; Wilgers Medical Consortium, Die Wilgers, Pretoria, South Africa; Interventional Cardiology Unit, Netcare Union Hospital, Alberton, South Africa; Mediclinic Bloemfontein, Bloemfontein, South Africa; South African Heart Association, Tygerberg, South Africa; Department of Cardiology, Faculty of Health Sciences, Steve Biko Academic Hospital and University of Pretoria, South Africa

**Keywords:** cardiology training in South Africa, cardiothoracic surgery training in South Africa

## Abstract

Over the past decades, South Africa has undergone rapid demographic changes, which have led to marked increases in specific cardiac disease categories, such as rheumatic heart disease (now predominantly presenting in young adults with advanced and symptomatic disease) and coronary artery disease (with rapidly increasing prevalence in middle age). The lack of screening facilities, delayed diagnosis and inadequate care at primary, secondary and tertiary levels have led to a large burden of patients with heart failure. This leads to suffering of the patients and substantial costs to society and the healthcare system.

In this position paper, the South African Heart Association (SA Heart) National Council members have summarised the current state of cardiology, cardiothoracic surgery and paediatric cardiology reigning in South Africa. Our report demonstrates that there has been minimal change in the number of successfully qualified specialists over the last decade and, therefore, a de facto decline per capita. We summarise the major gaps in training and possible interventions to transform the healthcare system, dealing with the colliding epidemic of communicable disease and the rapidly expanding epidemic of non-communicable disease, including cardiac disease.

## Why is it important to train specialists in cardiovascular care and heart health in South Africa?

The Global Burden of Disease study has highlighted that South Africa has an unacceptably high proportion of premature mortality^1^ and disability-adjusted life-years (DALYs) lost from cardiovascular disease. This is largely in part due to marked increases in hypertensive, rheumatic as well as ischaemic heart disease, and heart failure due to cardiomyopathy from 1990– 2010.^2^ Rapid demographic changes and adaptation to the so-called Western lifestyle, which includes low physical activity and high intake of processed high-caloric food, has led to more than one-third of the population being obese and hypertensive.^3^

In South Africa and sub-Saharan Africa (SSA), the spectrum and manifestation of cardiovascular disease is complex and markedly different compared to high-income countries, as rheumatic heart disease (RHD), tuberculous pericarditis and the cardiomyopathies remain common and often present at an advanced stage due to cardiac failure.^4^ Furthermore, over half of the patients hospitalised with heart failure are under 52 years of age.^5^ Patients with myocardial infarction are typically two decades younger than patients in the USA and Europe^6^ Table 1., and RHD becomes symptomatic in adulthood. The onset of serious heart disease before the sixth decade of life has important economic and other implications for South African society.^7^

**Table 1:** Comparison of age at first myocardial infarction

*Region*	*Medium age, women*	*Medium age, men*
Western Europe	68	61
Central and Eastern Europe	68	59
North America	64	58
South America and Mexico	65	69
Australia and New Zealand	66	58
Middle East	57	50
South Asia	60	52
Africa	56	52
China	67	60
South-east Asia and Japan	63	55
*Ethnic origin*		
European	68	59
Chinese	67	60
South Asian	60	50
Other Asian	63	55
Arab	57	52
Latin American	64	58
Black African	54	52
Coloured African	58	52
Other	63	53
Overall	65	56

A study from the Heart of Soweto cohort, reporting on the incidence and clinical characteristics of newly diagnosed RHD in adulthood from an urban African community, found an estimated incidence of new cases of RHD for those over 14 years of age to be in the region of 23.5 cases/100 000 per annum.^8^ Due to undetected RHD in the early years, many of those patients presented late, with left or right heart failure. Subsequently, one-quarter of this cohort of 344 cases needed valve replacement or repair within one year, with a further 26% being admitted for initial diagnosis of suspected bacterial endocarditis within 30 months.

The severity of disease was further corroborated in a recent study from 12 African countries, including South Africa, India and Yemen. Patients with RHD were young (median age 28 years), largely female and mostly severely affected.^9^ A further burden of late diagnosis of RHD is the fact that women often present with symptomatic RHD only when pregnant. A fouryear audit of cardiac disease in pregnancy in a South African hospital found an aetiology of 63.5% of RHD and 20.1% of prosthetic valvular heart disease (probably of RHD origin) among these women.^10^

A recent single-centre cohort study of 225 consecutive women presenting with cardiac disease in pregnancy at a dedicated cardiac disease in maternity clinic at Groote Schuur Hospital, Cape Town, highlighted the complex burden of symptomatic RHD (26%), congenital heart disease (32%) and severe cardiomyopathy (27%), among other cardiac conditions.^11^ Mortality occurred typically in the postpartum period beyond the standard date of recording maternal death, as also highlighted in a recent publication in the Lancet.^12^ The confidential inquiry into maternal deaths in South Africa reported that, of the 4 867 deaths reported over two years, 14% were due to hypertensive disorders, with another 8.8% due to medical and surgical conditions.^13^ Medical disorders, in particular cardiac disease complicating pregnancy, were the fourth most common cause of maternal death during pregnancy.

How well is South Africa prepared to transform our healthcare system to meet the demands of two colliding and interacting epidemics: a communicable disease epidemic of HIV/AIDS and tuberculosis, and a rapidly expanding second epidemic of non-communicable cardiovascular diseases?

## Training of doctors and healthcare personnel in South Africa

Between 2000 and 2012, the number of medical students enrolling per annum increased by 34%, with a major and deliberate demographic shift towards more female students and African blacks.^14^,^15^ Subsequently, the number of graduating doctors has increased by 18% in the same time period. However, the ratio of physicians per 1 000 population remained the same (0.77 in 2004 vs 0.76 in 2011) and is failing to keep up with the growth of the population.^15^

In response to the fact that the academic health workforce in South Africa is aging, numbers are shrinking, and there is a decline in clinical research capacity and output, two new research training tracks within the professional MB ChB programme have been created. These are the intercalated BSc (Med) Hons/MB ChB track and the integrated MB ChB/PhD track.^16^ Furthermore, the Ministry of Health has pledged to train 1 000 clinician PhDs though the National Health Scholars Programme over the next 10 years, providing scholarships equivalent to the salaries of health professionals employed by the Department of Health.^16^

## Cardiology training in South Africa is not keeping up with demand

**Fig. 1.**
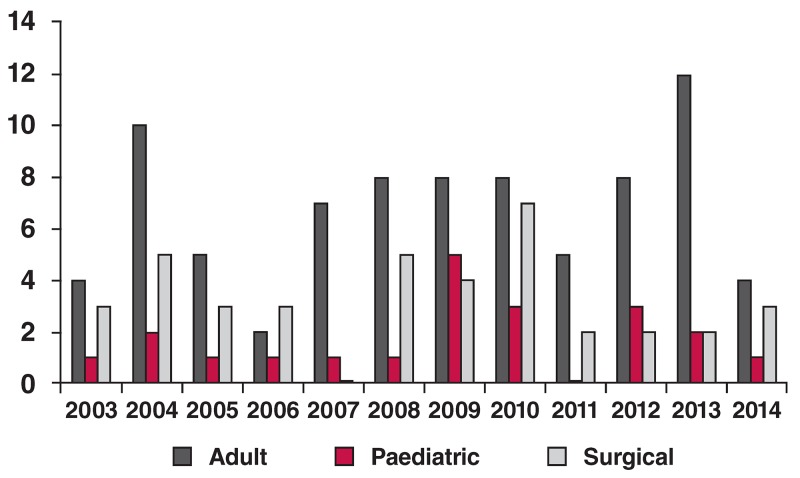
Number of cardiologists, paediatric cardiologists and cardiothoracic surgeons qualified in South Africa between 2003 and 2014.

What has changed in the training of cardiologists in the past decade? In South Africa, cardiology training is a three year subspecialist degree following the four-year training as a general physician (after being a house officer and having completed the community service time, bringing the total number of years for training to 15 years). (Fig. 1) highlights cardiologists successfully qualified per annum with minimal change over the last decade and, therefore, a de facto decline per capita.

The number of registered cardiologists in the country is currently about 200 for 52 million South Africans (one for 260 000). How does that compare with, for example, Brazil or one of the other BRICs countries with similar cardiovascular health issues to South Africa? Brazil has 8 000 board-certified specialists in cardiology for a population of 185 million (1:23 000) or 10 times more cardiologists and, even with that number, is not adequately equipped for the enormous cardiovascular challenges.^17^

If we use a conservative estimate based on the Brazilian numbers, we still require at least 2 000 cardiologists in this country. Furthermore, there is an unequal distribution of South African cardiologists servicing the private sector, as opposed to those servicing the public sector, where the greatest need for service delivery exists. In order to equalise or rectify this discrepancy, there needs to be ongoing political involvement in creating posts in the public service sector and equalisation of remuneration.

This has a number of serious consequences. There are inadequate or even no services at many tertiary and regional hospitals, as well as no pacing facilities in several provinces in the government set up. This is clearly linked to untimely death due to easily treatable medical conditions – patients may succumb from heart block without insertion of a pacemaker, have inadequate therapy without reperfusion with thrombolytic agents in acute ST-elevation myocardial infarction or suffer inadequate management of acute heart failure. Rheumatic heart disease is not diagnosed timeously due to poor access to advanced diagnostic facilities, such as echocardiography practiced by a cardiologist.

## Paediatric cardiology training in South Africa

The story of congenital heart disease is one of the major successes of medicine in the last 50 years, with the vast majority of congenital lesions now being amenable to surgery.^18^ However, the situation in SSA is startlingly different.^19^ There are only a handful of specialised cardiothoracic centres in SSA, and the majority of children requiring congenital heart surgery do not have access to these centres.^20^ Furthermore, cardiac catheterisation laboratories that are able to perform procedures such as ductal closures, pulmonary valvotomies and mitral valvuloplasties on children are also limited in SSA.

In South Africa, we appear fortunate in that we have the expertise to manage almost all of the congenital lesions, with the training institutions for surgeons and cardiologists consistently preforming over 300 cases in certain centres and over 1 500 per year in the country. However, the reality on the ground is that, as our primary healthcare services improve and awareness of congenital lesions increase, more patients will be referred with congenital heart disease requiring intervention. Despite many medical advances in the field, we remain critically understaffed, with increasing waiting lists and inadequate numbers of operations per year for our population.^21^,^22^

**Fig. 2.**
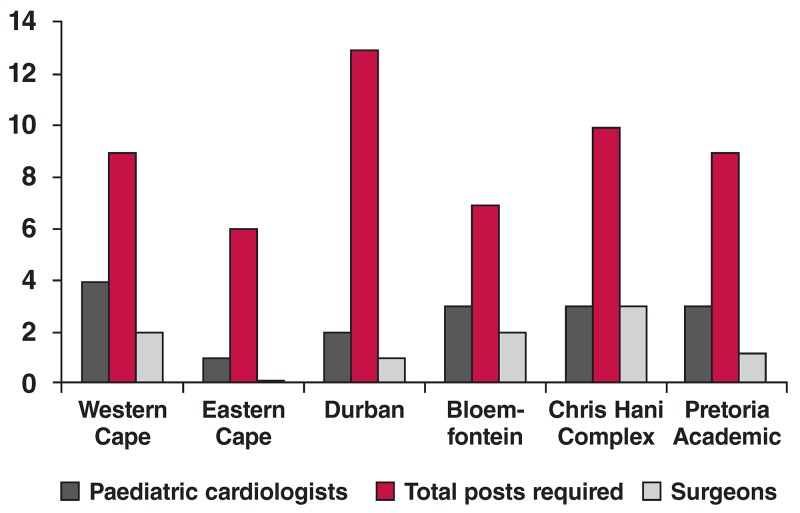
Consultant paediatric cardiology and cardiac surgery staff in the Public Service.

It is estimated that one paediatric cardiologist is required for every 500 000 population. If we use a conservative estimate based on half that number (one per million), we still require at least an additional 10 paediatric cardiologists in the public service in this country. Audits performed in 2010 and 2013 revealed that the number of paediatric cardiologists in the public service has increased by only one since 2010. In addition, as there is no subspecialist training for paediatric cardiothoracic surgeons, no significant increase had occurred in the number of children being operated on each year (Fig. 2).

Only two of the six national units consistently do over 150 operations per year. This has resulted in waiting lists in all the public service centres that exceed 100 patients, with many dying while waiting for surgery

A particular concern is the fact that several provinces do not have any regular paediatric cardiac services. This implies that referrals between provinces are the only option for these patients, which involves significant logistical, economic and transport difficulties. We know that certain critical congenital heart disease (CCHD) lesions are rarely seen in these provinces, suggesting early demise of those affected, without a definitive cardiac diagnosis.

The importance of paediatric cardiology training is not only for tertiary institutions but also to increase awareness among secondary and primary-level institutions. The inadequate number of public service paediatric cardiologists affects training and outreach programmes to our neighbouring provinces and countries.

Several novel approaches have been attempted to remedy this. The Walter Sisulu Centre of Africa previously attempted to fill this gap, the African Paediatric Fellowship Programme (APFP, http:// www.paediatrics.uct.ac.za/scah/apfp) serves to train paediatricians from across Africa, including paediatric cardiologists and surgeons, and collaborations between countries such as South Africa and Ethiopia have used task shifting to build capacity.23 Paediatric cardiac services in several provinces, such as Limpopo, rest on the shoulders of paediatricians with an interest in paediatric cardiology in order to diagnose patients, refer timeously and continue post-operative management and treatment.

## Cardiothoracic surgery training in South Africa

Currently, training in cardiothoracic surgery requires entry into a four-year programme post qualification. The four-year training programme encompasses a three-part examination in General Surgical Principles Part I, Intensive Care Principles Part II and Cardiothoracic Surgical Adult and Congenital Surgery Part III. Entry into the discipline is dictated by a single exit examination, which can be sat after acquiring Part I and II. The exit examination needs to be supported by a case load report, verified by the head of department prior to, or at the time of, sitting the Part III examination. Further requirements are competency in the practice of cardiothoracic surgery, which needs to be evaluated and confirmed by the head of department where the individual has undergone training.

To enable the country to satisfy its need for surgeons in the discipline, there are currently seven academic departments with a staff compliment of 28. Currently there are 21 surgeons-intraining at various residency levels.

The number of registered cardiothoracic surgeons in the country is 103. The need for cardiothoracic surgical expertise is estimated to be one surgeon per 800 000 population. Currently, the number of surgeons per population equals one per 4.5 million. Furthermore, there is an unequal distribution of surgeons servicing the private sector, as opposed to those servicing the public sector where the greatest need for service delivery exists. In order to equalise or rectify this discrepancy, there needs to be ongoing political involvement in equalisation of remuneration.

The current College of Cardiothoracic Surgeons of the College of Medicine of South Africa is in the process of reviewing the training period and instituting a recommendation and requirements for the training period to be extended to a total of six years.

The Society of Cardiothoracic Surgeons of South Africa is involved in coordinating additional training of residents by having established a Residents’ Forum in 2000. This Residents’ Forum has now been embellished by the involvement of the European Association of Cardiothoracic Surgeons education programme, which has contributed to this meeting in the past years. The Society is also currently involved in establishing an exchange programme between the Israeli Society and the South African Society, whereby a number of registrars and/or consultants will be exchanged on an annual basis in order to further enhance the training of South African surgeons and vice versa.

## The subspeciality of cardiac electrophysiology

The subspeciality of cardiac electrophysiology (EP) and pacing has become one of the more popular subspecialities in cardiology worldwide. However, EP in South Africa and SSA has long been considered to be a ‘niche’ subspeciality. Over the past 20 years, Groote Schuur Hospital in Cape Town has attracted several full-time EPs, and currently has the only full-time academic EP in South Africa. Fortunately, there are two part-time EPs performing sessions at Chris Hani Baragwanath Hospital in Johannesburg and Albert Luthuli Hospital and Grey’s Hospital in Durban and Pietermaritzburg, respectively.

The legacy of having a full-time EP service at Groote Schuur Hospital has stimulated interest in the field and has led to a further seven Groote Schuur Hospital cardiology registrars subspecialising in EP, mostly in North America and Europe, over the past 10 years. No training post for an aspiring EP exists in South Africa and all will need to perform an overseas fellowship (usually two years in duration). There are currently 13 CASSAaccredited EPs registered in South Africa, with a rough estimate of one EP per 21 million people in the public sector, compared to one EP per 800 000 people in the private sector.

It is not surprising that EP does not form a significant part of the core cardiology curriculum of cardiology training in South Africa. Cardiology registrars need to observe 15 EP cases to complete the logbook for the certificate in cardiology. This is inadequate to teach and understand the principles and practices of EP and does little to stimulate interest in the field. Most of the cardiology registrars outside of Cape Town observe cases in private hospitals around South Africa.

Cardiac pacing for bradyarrhythmias is considered a core skill in the training of cardiologists in South Africa. Cardiology registrars need to implant a minimum of 30 cardiac pacemakers (including five dual-chamber pacemakers) before being considered for the written and oral examination. The practical training of cardiac pacing at academic institutions is highly variable and mostly taught by general cardiologists. Many institutions are dependent on industry for device interrogation and troubleshooting.

The current implantation rate in South Africa is 60 per million people, which is much lower compared to European countries such as Germany, where the implantation rate is much higher. There is still a lack of cardiac pacing in four out of 11 provinces in South Africa. There is also a severe shortage of pacemaker implanters in the rest of SSA. In order to address this urgent need for cardiac pacing, the PASCAR Fellowship in Cardiac Pacing has been established. Doctors will be able to learn the principles and practices of cardiac pacing at Groote Schuur Hospital for a six-month period – the first fellow started in March 2016.

Implantable cardioverter defibrillators (ICDs) and biventricular pacemakers are limited in most academic institutions because of financial constraints and a lack of skilled expertise to implant them. Cardiology fellows need to observe 10 ICD and 10 biventricular implants for the logbook. Further additional training is often needed before cardiologists feel competent to implant them. CASSA has identified the need to improve the management and implantation of ICDs and has proposed an additional accreditation examination for non-EPs. Currently, all aspiring electrophysiologists need to seek overseas training, usually in North America or Europe. Compared to the United States, which has one EP per 127 500 people, it is estimated that South Africa needs a further 400 EPs for the equivalent population!

## The way forward

Having identified the profound deficiencies in the training of cardiologists, cardiothoracic surgeons and paediatric cardiologists in our country, the SA Heart Executive Committee, at three National Council face-to-face meetings held in 2015, identified the need to prepare a position paper summarising the facts and exact training deficiencies in South Africa over the past decade. It was generally felt that specialists in academia and private practice need to work more closely together and endeavour to engage with members of the Departments of Health and Education. In addition, the pharmaceutical, device and private hospital groups should be encouraged to sponsor more training posts in academic institutions.

To facilitate earlier disease detection (e.g. RHD in young adults, including pregnant women) in a country and continent with so few specialists per capita, capacity for screening will have to be increased. This could be achieved by training a larger group of healthcare workers in the appropriate use of simple, hand-held ultrasound devices. The screening for relevant diseases requiring referral to secondary and tertiary facilities could then, to some extent, be performed by general practitioners and medical technologists.^24^

In cases of women presenting with cardiac symptoms in pregnancy, the obstetricians could receive training in screening for cardiac diseases, as is currently already in practice via a dedicated clinic at Groote Schuur Hospital. Obstetricians have good ultrasound skills and the detection of turbulence over a rheumatic valve, a myopathic heart or a pericardial effusion would lead to quicker referral to cardiologists or cardiothoracic surgery for appropriate intervention.

SA Heart is cognisant of the fact that the health authorities face tremendous challenges. Mismanagement and a burgeoning layer of bureaucracy have had a catastrophic effect on the delivery of healthcare. This deficiency is evident at all levels, including the training of nurses and specialists, and the provision of an adequate infrastructure for the practice of an acceptable level of medicine. Fundamental to this issue is the failure by the government to recognise that academic and tertiary health facilities are not a burden to the economy but underpin a strong and effective health system. In particular, provincial government has abrogated its responsibility to support and promote tertiary medicine, which has culminated in poor service delivery to the indigent patient and plummeting morale among the academic community.

In the setting of very limited resources and previously poorly investigated sectors of the population, it is very important that cardiologists are trained to deal with a broader range of disorders so that they are recognised and appropriately managed away from the few teaching hospitals. In such hospitals, it is important to have good support for emerging subspecialities within cardiology to integrate service, teaching and research that is relevant to this country. Specific areas that need attention include cardiology in pregnancy, heritable disorders predisposing to cardiomyopathy and arrhythmia, and metabolic disorders including especially familial hypercholesterolaemia, in which much progress in treatment has been made. Rumours that management of tertiary hospitals will be taken over by the national government seem promising but unlikely to occur any time soon.

**Fig. 3.**
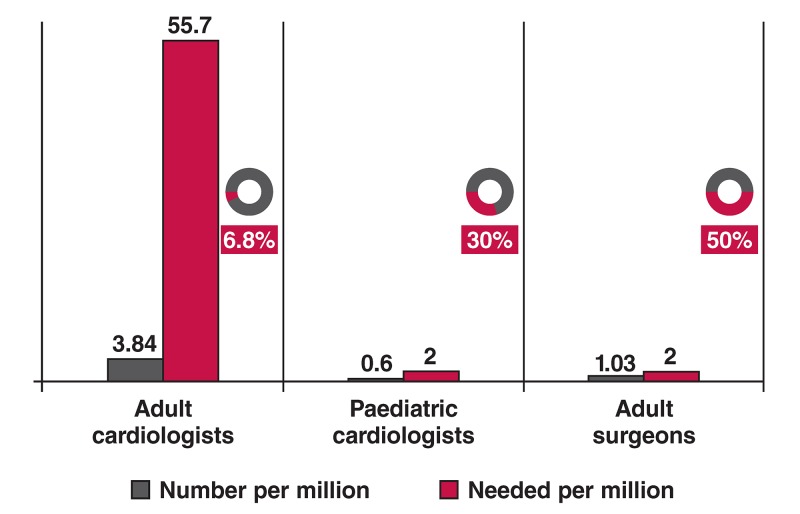
Registered specialists in South Africa versus number of specialists needed per million population.

The committee has summarised gaps and possible interventions in Table 2.. In addition, (Fig. 3) depicts the number of specialists registered currently in South Africa versus the specialists needed to serve the country.

**Table 2:** Summary of the gaps identified and suggested next steps

*Gaps identified*	*Suggested next steps*
Low rates of graduates from health professional schools due to inadequate training posts	Create more posts for cardiovascular academics, promote career development and other incentives for teaching roles
Inadequate pool of trained cardiovascular specialists	The establishment of a private training centre for cardiologists following the same curriculum they follow in the state, based at a centre of excellence
Internal and external brain drain	Close engagement of specialists in academic hospitals and private practice
Specialists not used optimally, considering low number of specialists in South Africa and therefore late or inappropriate referral from the community level	Use of non-physician technicians, medical officers in the use of handheld echocardiography for early cardiac disease detection, facilitating early referral to cardiologist/cardiothoracic surgeon
Specific training needs in cardiology in pregnancy, heritable disorders predisposing to cardiomyopathy and arrhythmia, and metabolic disorders, including especially familial hypercholesterolaemia	Training of obstetricians in the detection of cardiac disease, facilitating early referral to cardiologist/cardiothoracic surgeon.
	Registrar rotation through special clinics
*Health system weaknesses in CVD area*	
Lack of strategies for increased specialist training for cardiovascular disease in South Africa	Closer engagement with Departments of Health and Education to increase training posts
Insufficient epidemiological data on CVD and its medical and surgical management in South Africa	Improve science and technology infrastructure, acquiring better epidemiological data on CVD as part of health system-strengthening strategies
Overall low CVD scientific output, making healthcare planning difficult	Progressive increase in the percentage of GDP allocated to research and development, better recognition of the role of the clinician–scientist and subsequent increase in scientific output related to cardiac disease
Health policy decision makers and cardiovascular specialist inertia to increase training opportunities	Invest in regulation that promotes public–private partnerships on research
Low investment in research and development infrastructure and lack of science and technology culture	Facilitate translational research
	Facilitate training in cardiovascular research in South Africa and collaborations with international research entities

## Conclusion

SA Heart is deeply concerned that the government has reduced and frozen training posts, progressively whittled down the number of tertiary hospital beds, and has failed to provide an environment conducive to the delivery of healthcare that would comply with internationally acceptable standards.
